# A Treatise on Pyrosis Idiopathica, Waterbrash, &c.

**Published:** 1841-10-01

**Authors:** 


### A TREATISE ON PYROSIS IDIOPATHICA, OR WATER BRASH, AS CONTRASTED WITH CERTAIN FORMS OF INDIGESTION AND OF ORGANIC LESIONS OF THE ABDOMINAL ORGANS; TOGETHER WITH THE REMEDIES, DIETETIC AND MEDICINAL. By *Thomas West*, M.D. &c. &c.

IT is perhaps quite true, that if a man were to sit down to write upon "Dyspepsia" at length, he would have almost as much to do, as if he had undertaken to unfold the mysteries of that perplexing class of disorders, to which the name of "hysteria" is generally applied. In the present day, therefore, when folios are out of fashion, it may be advisable for an author to content himself with the mere exposition of one of its forms, as affording the only chance of getting to the end of his subject, within anything like a reasonable number of pages; but still this unnatural division of diseases, by the consideration of one symptom, severed from those of which it forms a necessary part, has manifest practical disadvantages, not the least of which is, that the writer is imperceptibly led, not only to ascribe to it an undue importance with respect to the others, but even to exalt it so far above them, as to come at length to look upon it as the essential cause of the disease itself. In the treatise now under notice, however, the author may suppose that he has escaped from this error, because he altogether denies that the disease of which he treats is to be considered as a symptomatic one: his very title is "*pyrosis idiopathica,*" and the whole train of his reasoning is directed to prove that it is a disorder per se, quite distinct from the various forms of dyspepsia, which, according to him, is an "organic malady," always complicated with disturbance in other viscera besides the stomach. It is probable, however, that persons who, like ourselves, have no favourite theory to support, will look upon it as another instance of an attempt to magnify a mere form of disease into a separate, or essential one.

Pyrosis, by which is meant a burning pain in the stomach, attended with copious eructation of a watery insipid fluid, known in Scotland by the name of "water brash," and in some parts of England by that of "black water," has been described by nearly all writers as a frequent symptom or attendant, in a greater or less degree, of one or other of the forms of dyspepsia: it has been duly noticed in this practical relation, and certainly seems to have received its proper degree of attention, as a part of a complicated malady: therefore when our author complains (*vide p.* 29) that it has been but very insufficiently described by previous writers, he must mean that it has not been described to his own liking, or in accordance with his views of its nature, which is certainly the case, and which will probably continue to be so; since it is not every one who will be brought to look upon pyrosis as "dropsy of an open cavity," resulting from impaired innervation, produced by a regular current of "depressing and poisonous influences," from without to within.

Still the work is far from being devoid of interest or of usefulness, especially in the remarks relating to the dietetic causes of the malady: the style is somewhat rambling, and disfigured here and there by the use of oddly latinised words, which have no very precise meaning: added to this, there is a kind of running commentary, throughout the book, on other men's opinions, which renders it no easy matter to get at the author's own; which however, as we think are worth noticing, we shall proceed to give an analysis of.

The Treatise, though not divided into chapters, or parts, successively explains the pathological nature—the means of diagnosis—the post-mortem degenerations—and the treatment of pyrosis idiopathica, and we shall follow this order in our remarks upon it.

According to our author, the disease in question is a serous flux from the terminal exhalants of the stomach, arising from nervous debility, as well general as local; and which debility is induced by peculiar causes to be afterwards described. It is however always advisable to give the author's own words on these fundamental points, particularly when it happens that his language is not very plain: it is rather difficult however to find a paragraph, in any of the earlier pages, which will be sufficiently explicit to give a correct idea of his opinions; but at p. 52, we have the following, which appears to embrace the essentials of his theory, which he is there attempting to illustrate by comparing pyrosis with cholera.

"In pyrosis the skin and stomach are gradually affected, the innervation impaired, the circulation rendered languid and defective, the digestion feeble and imperfect, the animal spirits gradually depressed, the temperature and temperative principle manifestly at fault, the containing power of the blood-vessels loosened, the serous portion of the blood infiltrated, and considerable portions of the more watery part of it discharged by paroxysms." 52.

Now when we add to this that our author supposes, that in pyrosis idiopathica, the fluid is secreted as well from the fauces and œsophagus as from the stomach, and that, from its collection there, the disorder ought to be looked upon as "dropsy of an open cavity," we have presented in a small compass the whole of his views on the matter; but before we go further, let the author again speak for himself.

"Pyrosis then, in this our view, is only an aggravation of one of the common symptoms of derangement of the stomach. The pathological condition of the organ is one of great debility. For what is the affection but a dropsy of an open cavity? What is its proximate cause but an exhausted and disturbed state of nervous energy?" 3.

Even the writer himself, immediately after penning this passage, appears to have some misgivings as to the manner in which such an opinion may be received: thus he says—

"In asserting that it is a dropsy of an open cavity, I am aware that it is a secretion of a mucous membrane, and duly bear in mind all the differences of the two tissues. Nevertheless, for practical purposes, I consider the analogy to be sufficiently strong; and I will go further and add, that they who are in years, and pyrotic, will not be long without dropsy, unless the condition of stomach be improved." 4.

Now for our own parts we cannot see the analogy at all; the organization of the structures referred to is so different: the mode in which diseases commence and terminate in them is so different, that even if we were inclined to admit that the pyrotic fluid is a mere bland serous exhalation, we could not compare it with the albuminous contents of a shut sac: besides few now-a-days look upon real dropsical effusions as the result of atony of the secreting part, but rather as the effect of congestion in the circulating system. In our own opinion, it would be as rational to call a mucous catarrh from the lungs a dropsy, as to do so in the case of pyrosis. Neither do we at all recollect to have observed any disposition in old "pyrotic patients" to become dropsical; on the contrary, they generally incline to the reverse, being for the most part dry, shrivelled, and emaciated; but when a man has once started a theory, he is generally resolved to maintain it "coute qui coute," and in doing so, often endeavours to elucidate it, by reference to facts which do not seem to bear at all upon the question: thus, we do not see what light is thrown upon our author's views of pyrosis by the following sentence:—

"Nor are these the only morbid secretions of the same serous vessels. In some instances they secrete an acid liquor sufficiently corrosive to excoriate the tongue as it passes over it. I suspect that those cases of ventricular perforation which have in the north of England been made subjects of jurisprudent controversy, are of this nature. We all recollect how warm was the dispute of certain eminent physiologists on this matter. The discrepant evidence given by them before a judge and jury was reiterated in pamphlets and rejoinders. In my humble opinion it was a mere waste of words, and I deferentially assign these my reasons. 6.

We must refer our readers to the work itself for these reasons, begging to remind the author that, independently of John Hunter, Spallanzani, Adams, Burns and others, have proved the gastric juice to have the power of dissolving the coats of the stomach, after death, so as to produce a perforation of them: and that more recently Carswell has put the matter beyond all doubt: let us close this part of the subject with shewing what our author believes the gastric juice to be.

"I believe pyrosis to be a disease of serous vessels destined to secrete a fluid, which, when healthily acted upon by the other fluids, viz. the salivary, the oral, the pharyngeal, and the glandular muco-acid juices of the stomach, becomes gastric juice; and I believe in the existence of healthy gastric juice in no other sense but this." 33.

Then there follows a kind of summary of the opinions of other writers on this disease, the grand object of which is, to pick out those which appear to confirm the author's theory: the whole of this however conveys no exact information to our minds, being full of false analogies and a mode of arguing the matter which sometimes approaches to the ridiculous: take the following example.

"Cold and damp *ab externo* induce dropsy. The continued application of various badly-selected articles of food to the surface of the stomach slowly poisons the surface, affects the innervation, and induces pyrosis. Cold and damp *ab externo* favour pyrosis. Bury a man in salt to his neck for a few hours, and you will have dropsy." 25.

We really cannot say what a man might have after such treatment, but think it likely that he might be the worse for it, in some way or other.

On the matter of diagnosis, our author is not very minute, nor indeed would it have been necessary for him to be so, unless he had been endeavouring to establish a new disease, since there cannot be much difficulty in deciding upon the existence of pyrosis. In reality, he nowhere gives anything like a connected history of the symptoms, but contents himself with expounding those of the diseases with which he says pyrosis is apt to be confounded, and then labours to shew a difference between them. So long, then, as the comparison is confined to cancer of the cardiac or pyloric orifices of the stomach, to organic disease of the pancreas, or to symptomatic pyrosis from pregnancy, the distinction is evident enough; but gastrorrhœa (or, as the author proposes to call it, "*stomach gleet,*") presents, as may be imagined, a serious obstacle to the maintenance of his views on the peculiar nature of pyrosis, for it is unquestionably the same disease in excess, and is described as such under various names, by all writers on the subject. Our author however is able to detect important differences between them, one being that in gastrorrhœa, there is burning pain in the stomach prior to the "rumination" of the fluid, which is hot and acrid, whereas in pyrosis there is no pain, and the secretion is insipid; another being that, in the former complaint, the discharge is from the "follicular glands of the stomach," and, in the latter, results from disease of the "terminal exhalants;" whilst a third is a mere general distinction, to the effect that "pyrosis is a disease of *serous* temperaments; stomach gleet of pituitous." To say that pyrosis is not attended with burning pain is to have no regard for the very name by which it is distinguished, or for one of its most prominent features; and, as respects the sensible characters of the secretion, it is well known that the pyrotic fluid varies in different cases, and even in the same persons at different times. It is nearly always alkaline; occasionally acid; generally insipid, but sometimes hot and acrid: therefore it cannot be allowed to have the uniform qualities which the present author wishes to assign to it.

It is all very well to assume it to be an atonic effusion from "the serous structure of the stomach, in lax and lymphatic temperaments," but this is at the best but a loose way of talking, which conveys no precise idea to those who are not obliged to imagine particular pathological conditions for the confirmation of particular views. The same may be said of the physiological opinions with respect to the filtering powers of the stomach, by which noxious agents are allowed to "percolate" its coats, and so to "depurate the blood," by casting off certain excrementitious matter in the same way as is effected in the kidneys by the secretion of urine. Those who want to know how this comes to pass, and what connection it has with pyrosis, may read all about it at p. 38.

Of the "post-mortem degenerations," as our author terms it, we have little or nothing, for he declares that "it is difficult to appreciate the pathological phenomena," and again, that "these must be rather a matter of conjecture than of history." Still, after so much had been said of the "pathological condition" of the parts concerned in this affection, we were not prepared for such a confession; we quite expected to be told that there was something more than "a mucous surface unusually pale," for as the disorder had been considered in an unusual point of view, with a special reference to certain essential organic changes, we looked for a corresponding account of the morbid appearances, as the groundwork on which the theory was built; all we have, however, on this head is as follows:—

"The appearances of the stomach in true water-brash would only indicate a state of adynamic dropsy of that organ, just as a pale skin in cold sweats indicates a similar condition of the cutaneous tissue." 46.

We now come to that part, wherein the chief practical object of the author is expressed, consisting of an exposition of the dietetic causes of the malady; and the subject is ushered in by the following paragraph in italics:—

"*Chemical and chemico-vital remarks on the non-azotic or not sufficiently azotic constitution of the aliment usually consumed by the population in districts where Pyrosis is frequent.*" 49.

These chemical and chemico-vital remarks then are intended to prove, "that the steadily and constantly depressing influence of a humid cold atmosphere, on the periphery of the circulation, and slight but successive and daily repeated poisonous impressions made upon the internal digestive tube, are the joint agents which induce this malady."—(See p. 49.)

With this theory for the basis of his remarks, the author runs on through two dozen pages, occupied in shewing how these causes combined operate upon the "serous structure of the stomach," and the system generally, so as to produce pyrosis: and that the poor, whose diet is principally farinaceous during many months of the year, especially in particular localities, and who are necessarily exposed to the depressing influences of cold and damp, are found to suffer from it in proportion. The prevalence of dyspeptic disorders, among the poorer classes, is certainly very remarkable, and possibly would not be anticipated by those who have been in the habit of ascribing them to a too luxurious system of diet; there is probably then a good deal of truth in our author's remarks on this subject, although there can be little doubt that he carries the thing somewhat too far. *We* may believe, that a state of parts approaching to chronic gastritis, is the real pathological condition in the majority of these dyspeptic cases; but the exciting cause may reasonably be surmised to have its origin in unsuitable food, combined with the absence of those domestic comforts, the want of which are alone a fertile source of disease. In fact, all the forms of dyspepsia have always been set down, as occasionally arising from this cause, and we think it scarcely to be doubted (particularly by those who have practised among agricultural poor) that the constant use of aliment, not sufficiently nutritious or diversified, that is to say, of course, badly prepared farinaceous food, only varied at long intervals, by the most indigestible of the animal substances, is the great cause of the prevalence of these disorders among them. Whether, however, this kind of diet produces essentially the kind of pyrosis which is the subject of our author's treatise, is quite another question, and we must first admit the accuracy of his views upon it before we can agree that it is so. It is to be borne in mind, however, that the author attributes the disease to certain injurious impressions upon the external cutaneous surface, as well as to the direct application of poisonous agents from within : it is the union of cold and damp with this poverty of diet, that induces the disorder, and it is for this opinion that the author claims the merit of originality.

"I scarcely know of any writer, foreign or domestic, who has not attributed the prevalence of the malady to a poverty of diet, although most of them have overlooked the fact, that a mere farinaceous diet in a tropical climate is not sufficient to produce the disease to any considerable extent." 54.

Again, he says—

"Let it be lawful to me to speak the truth without presumption. What assistance does the medical literature of Greece and Rome furnish to me in my investigation of this distressing malady? Absolutely none ; and manifestly because such genial climates were not calculated to produce it." 75.

Yet the books of the great Roman physician are full of peptic precepts, and if we recollect rightly, there is a chapter also, "*de Cardiacis*," where something like a hint of our author's own theory is given.

The treatment recommended by him is for the most part dietetic, and since he insists that his peculiar form of pyrosis is essentially a malady of the starving poor, he urges the wealthy and benevolent to exert themselves in procuring for them a regular and sufficient supply of good animalised food, with warm cottages, and those general comforts that will ensure them a permanent relief from mental anxiety! This would indeed be desirable under any circumstances; but, unless the golden age were to return, we do not see how it is to be accomplished : let us hope then that the evil may be remedied without any such miraculous interposition being necessary.

We quite think that it is the bounden duty of medical men, to represent in a proper manner to the proper authorities, the existence of anything which is ascertained to be injurious to the health of the community: but such representation should not be founded on mere speculation, and the plan suggested for the amendment of the evil ought at least to be of such a kind, as will admit of a practical application ; as society is at present constituted, however, the recommendation of Dr. West could not be carried out, even if it were generally looked upon as judicious.

According to our author, the treatment to be pursued is based on the following indications :—

"1st. To provide azotic food in order that the blood of the patient may be more highly animalized.

2nd. To increase steadily the vigour and heat of the circulating system, especially in the capillaries of the surface.

3rd. To give new tone and fresh impulse to the absorbent system." 105.

During the discussion of these indications, the author very absurdly inveighs against the apathy exhibited by the generality of town doctors, or those who practise exclusively among the rich, in relation to this disease; he declares over and over again, that such gentlemen know nothing about it, or if they do, that they have neglected to do anything towards its alleviation, because it happens to be a disorder, which is not met with among their favoured class of patients; thus, speaking of Sydenham, he says—

"Not one word has he placed on record concerning this malady. And why? He lived in our metropolis. He never practised any where else; and, like many of our modern great ones, he delighted in narrating cases commencing with 'Vir nobilis,' 'Comes,' 'Mulier magni nominis,' &c., &c., shewing that in the sphere of his practice he had but little chance of seeing an affection which exclusively belongs to the badly fed and miserably paid rustic, or to the provincial labourer." 78.

Then, a page or two further on, he launches forth into a style of invective which is altogether inadmissible in sober argument: for instance—

"It is not to authors (however great their talent) who have avowedly written for the rich and lazy, that we can look with any well-grounded expectation of suitable admonitions to lessen the miseries of patients suffering under this species of malady, for with some few exceptions, as an idiopathic disease it belongs exclusively to the poor." 80.

As a matter of course the guardians of the poor come in for their share of animadversion; we wish it was quite certain that they never deserved it. The author is speaking of the public officers of health, who existed in ancient Rome, under the name of "Ædiles Cercules," and laments that, in our days, their very important duties are usurped by men who are very incompetent to perform them; he then proceeds:—

"In our time, guardians, committee-men, or overseers claim to decide on the laws of dietetics, although their knowledge of the human frame barely extends beyond a vague idea that man is a tube of some sort open at both extremities. It has been argued by the friends of such a system that the medical officer has the privilege of presenting to a board of guardians his recommendations for the occasional introduction of such articles of nutriment as may to him seem best suited to meet the occasional exigencies of disease. It is not quite so evident that he has the moral certainty of seeing his recommendation carried into effect." 100.

From all that has been said then, it may perhaps be guessed that Dr. West assigns very little efficacy to the mere administration of medicines in this disease: but it may again be as well to let the author speak for himself on this point.

"We may try to rectify secretions, to brace up and fortify the stomach, but unless we can supply good wholesome food, animal and vegetable, together with warm clothing, a dry habitation, and the peace of mind which is the attendant upon a cheerful confidence, or at least a good hope, of a continuance of a comfortable 'daily bread,' all our measures will fall short of relief, and our pills and potions, our chemicals and our galenicals, will be a miserable mockery, a wretched satire upon that half science and half art which Cicero denominated 'God's second cause of health.'" 79.

After such a tirade, we should imagine that the author would not condescend to mention any mere medical remedies; and in fact he speaks slightingly of most of those which have long been acknowledged to have considerable power over the disease. Thus, according to him, bismuth is of no use whatever excepting in spasmodic dyspepsia; opium, "for true pyrosis as defined and explained in our account of it is decidedly injurious, rarely palliative, and never capable of effecting a cure." (p. 101.) Blood-letting in any shape, "in the popular affection of which we treat is decidedly mischievous," (p. 101), whilst nux vomica he will not incur the responsibility of recommending, but declares that, unaided by proper diet, it is good for nothing, and with it is quite unnecessary. Nevertheless in an "Appendix Medicaminum" he gives eight formulæ which he thinks merits recommendation; and as well as these, bark, quinine in sherry, warm balsams, and "spiced port wine," are favourably mentioned, though none even of these have any permanently curative powers unless assisted by a duly azotized diet.

The mineral acids are not even mentioned, neither is there much more than a passing allusion to hydrocyanic acid, or to creosote, all of which we would advise our author to try: and as a substitute for formula No. 2, which consists of nine different ingredients, we venture to recommend the following to his notice:

℞. Liquoris Calcis, ℥vij.

Spt. Ammon. Aromat. ʒss.

Tinct. Hyoscyami, ʒss.

Magn. Carbonat. gr. xx. M.

Haustus—bis quotidie secundus.

With this our remarks must close, wishing however, that the first parts of Dr. West's treatise had been more practical, and the last part more temperately written.

